# Prevalence and genetic characterization of *Cryptosporidium* spp. and *Enterocytozoon bieneusi* in horses in central and western Inner Mongolia, China

**DOI:** 10.3389/fvets.2026.1988226

**Published:** 2026-10-30

**Authors:** Yaning Li, Jingwei Quan, Junyan Li, Xiaoping Luo, Ziran Mo, Qian Zhang, Feng Li, Yabo Zhao, Xuerou Chen, Xiaoyue Du, Gaowa Gong

**Affiliations:** 1 Key Laboratory of Grass-Feeding Livestock Healthy Breeding and Livestock Product Quality Control, Veterinary Research Institute, Inner Mongolia Academy of Agricultural and Animal Husbandry Sciences, Hohhot, China; 2 College of Life Sciences, Inner Mongolia University, Hohhot, China

**Keywords:** *Cryptosporidium* spp., *E. bieneusi*, genotype, haplotype, horse

## Abstract

**Background:**

*Cryptosporidium* spp. and *Enterocytozoon bieneusi* are important enteric pathogens infecting humans and a wide range of animal hosts, including horses, and some species and genotypes have zoonotic significance. However, information on their prevalence and genetic characteristics in horses in Inner Mongolia remains limited. This study investigated the prevalence, species/genotype composition, and genetic diversity of *Cryptosporidium* spp. and *E. bieneusi* in horses from central and western Inner Mongolia.

**Methods:**

From May to September 2025, 325 horse fecal samples were collected from Otog Banner, Zhenglan Banner, and Abaga Banner, Inner Mongolia, China. *Cryptosporidium* spp. and *E. bieneusi* were detected by nested PCR targeting the SSU rRNA gene and ITS region, respectively. *C. hominis*-positive samples were further subtyped by *gp60* amplification. Phylogenetic and haplotype analyses were performed to assess genetic relationships and diversity, and prevalence was compared among geographic, age, and health-status groups.

**Results:**

The overall prevalence of *Cryptosporidium* spp. and *E. bieneusi* was 2.8% (9/325) and 1.5% (5/325), respectively. Two *Cryptosporidium* species were identified: *C. hominis* (55.6%, 5/9) and *C. parvum* (44.4%, 4/9). All *C. hominis* isolates belonged to the Ik subtype family, including IkA18G1 and IkA19G1. *Cryptosporidium* prevalence was significantly higher in foals than in adult horses (11.5% vs. 1.1%, *p* < 0.05) and in diarrheic than non-diarrheic horses (16.1% vs. 1.4%, *p* < 0.05). All *E. bieneusi*-sequences were identified as genotype horse1 and clustered within Group 1. Phylogenetic and haplotype analyses showed that the *Cryptosporidium* sequences were closely related to previously reported equid-derived sequences from China. *C. hominis* exhibited genetic diversity, whereas the *E. bieneusi* horse1 genotype showed a relatively conserved genetic structure.

**Conclusion:**

This study provides new molecular epidemiological and genetic insights into *Cryptosporidium* spp. and *E. bieneusi* in horses in central and western Inner Mongolia, including, the first regional report of the *C. hominis* gp60 subtype IkA19G1. The higher *Cryptosporidium* prevalence in foals and diarrheic horses highlights these groups as priorities for surveillance. These findings provide baseline information for future molecular surveillance and assessment of potential public health risks associated with these enteric pathogens in horses in this important horse-breeding region of China.

## 1 Introduction

*Cryptosporidium* spp. and *Enterocytozoon bieneusi* are two important zoonotic enteric pathogens that can infect a wide range of hosts, including humans, livestock, and wildlife (1–4). Both pathogens are primarily transmitted via the fecal–oral route and can cause diarrhea and malabsorption, with particularly severe consequences in children and immunocompromised individuals (5). In livestock, infection can lead to weight loss and impaired growth and development, and severe cases may result in mortality, particularly in young animals (6). Horses and donkeys are widely used for agricultural production, transportation, food production, and sporting and recreational activities, resulting in frequent close contact with humans (7). Multiple *Cryptosporidium* species and *E. bieneusi* genotypes have been identified in horses from different regions of China, some of which have also been reported in humans, suggesting a potential risk of zoonotic transmission (7–9). Therefore, molecular epidemiological investigations of *Cryptosporidium* spp. and *E. bieneusi* in horses are essential for determining their prevalence, species composition, and genotype distribution, as well as for assessing zoonotic risks and developing targeted prevention and control measures.

Inner Mongolia is one of the major horse-breeding regions in China, where the horse industry plays an important role in the local livestock economy (8). In 2018, the horse population in Inner Mongolia reached 848,000, ranking first in China.^1^ Previous studies have detected *Cryptosporidium* spp. and *E. bieneusi* in horses in Inner Mongolia, indicating that these enteric pathogens are circulating in local horse populations (8, 9). However, previous surveys were limited by relatively small sample sizes and restricted geographic coverage, with sampling conducted mainly in Hohhot and Hailar, and therefore may not fully reflect the epidemiological characteristics of *Cryptosporidium* spp. and *E. bieneusi* in horse populations across different regions of Inner Mongolia. Therefore, broader and more systematic investigations are needed to characterize the prevalence, species composition, and genotype distribution of these two pathogens, thereby providing baseline data for understanding their regional epidemiological patterns and assessing their potential zoonotic risks.

In the present study, horse populations in Xilingol League and Ordos City, Inner Mongolia, were surveyed for *Cryptosporidium* spp. and *E. bieneusi* using nested PCR and sequence analysis. *Cryptosporidium* species and *E. bieneusi* genotypes were identified, and their genetic characteristics were further evaluated by phylogenetic and haplotype network analyses. The present study aimed to systematically investigate the prevalence, molecular characteristics, and genetic diversity of *Cryptosporidium* spp. and *E. bieneusi* in horses in central and western Inner Mongolia by integrating epidemiological investigation with molecular typing and genetic analyses, thereby providing baseline data for assessing their potential zoonotic risks and developing targeted prevention and control measures.

## 2 Materials and methods

### 2.1 Sample collection

A cross-sectional survey of enteric pathogens in horses was conducted from May to September 2025 in Otog Banner, Zhenglan Banner, and Abaga Banner, Inner Mongolia Autonomous Region, China. Sampling sites were selected based on local horse distribution, breeding scale, and sampling accessibility (Figure 1). A total of 325 fecal samples were collected, with approximately 20 g collected from each animal. Among them, 60, 121, and 144 samples were obtained from Otog Banner, Zhenglan Banner, and Abaga Banner, respectively. According to clinical status recorded at the time of sampling, 31 fecal samples were collected from diarrheic horses, while 294 samples were randomly collected from healthy horses. Based on age classification, 52 samples were collected from foals (≤1 year old) and 273 samples from adult horses (>1 year old). All samples were immediately transferred into sterile fecal collection tubes, preserved in 2.5% potassium dichromate, and stored at 4 °C until further analysis.

**Figure 1:**
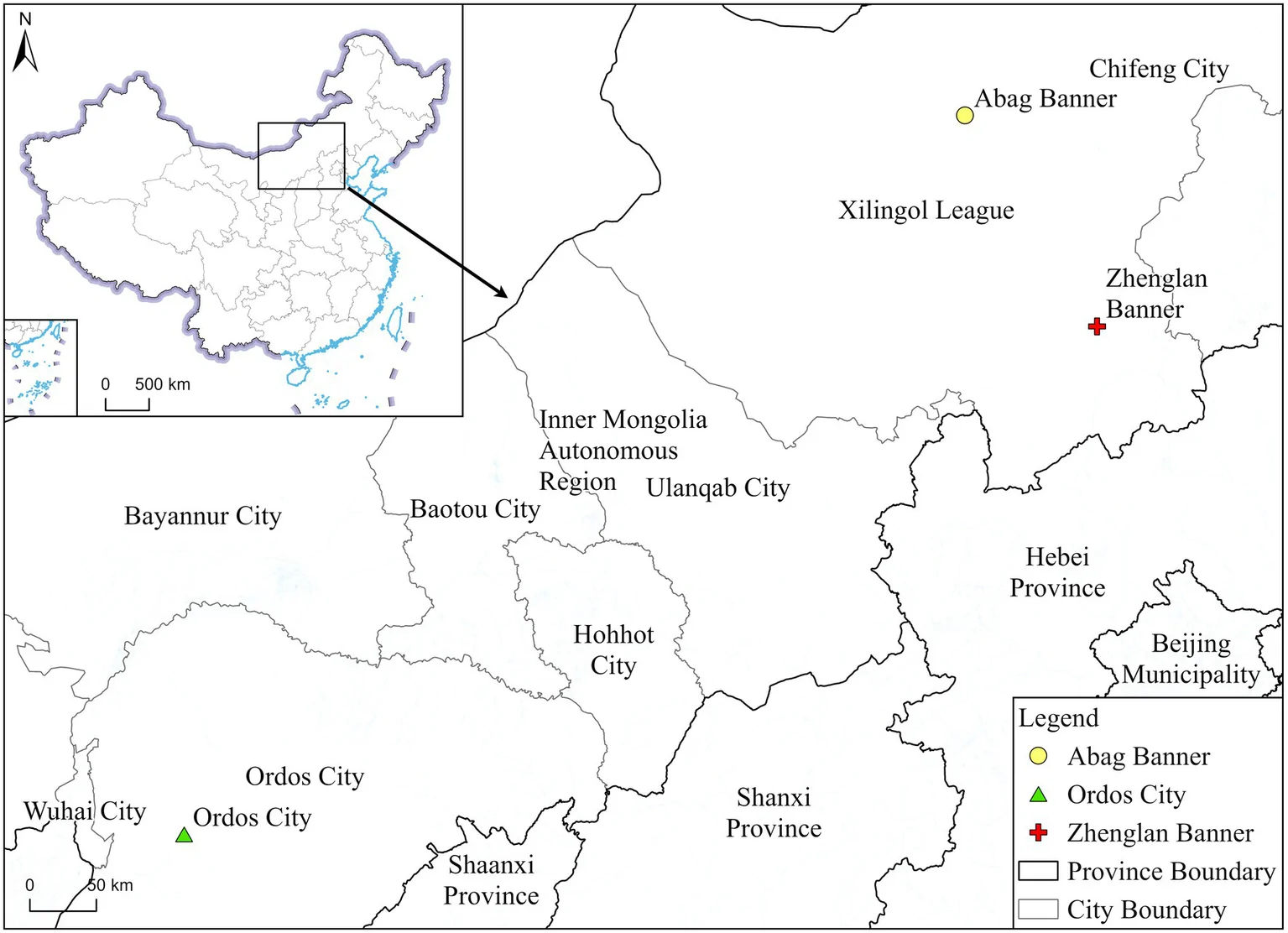
Geographic map of the sampling locations in Inner Mongolia.

### 2.2 DNA extraction and PCR amplification

The collected fecal samples were thoroughly resuspended in sterile distilled water and centrifuged at 1,500 × g for 10 min at room temperature. After discarding the supernatant, the washing step was repeated three times to remove residual potassium dichromate. Approximately 500 mg of each fecal sample was used for total DNA extraction with the FastDNA Spin Kit for Stool (MP Biomedicals, USA), according to the manufacturer’s instructions. The extracted DNA was stored at −20 °C until further analysis.

A nested PCR assay targeting the SSU rRNA gene was used to detect *Cryptosporidium* spp. (1). Primer pairs CF2/CR2 and CF1/CR1 were used for the first- and second-round amplifications, respectively. The cycling conditions were as follows: initial denaturation at 95 °C for 5 min; 35 cycles of denaturation at 95 °C for 30 s, annealing at 55 °C for 30 s, and extension at 72 °C for 30 s; followed by a final extension at 72 °C for 7 min (Table 1). Subsequently, an approximately 830 bp fragment of the *gp60* gene was amplified by nested PCR to determine the subtypes of *C. parvum* and *C. hominis* (10). Primer pairs GP60F1/GP60R1 and GP60F2/GP60R2 were used for the first- and second-round amplifications, respectively. The cycling conditions were as follows: initial denaturation at 95 °C for 5 min; 35 cycles of denaturation at 95 °C for 45 s, annealing at 55 °C for 45 s, and extension at 72 °C for 90 s; followed by a final extension at 72 °C for 7 min (Table 1). *E. bieneusi* was detected by nested PCR targeting a partial fragment of the internal transcribed spacer (ITS) region. Primer pairs ITSF1/ITSR1 and ITSF2/ITSR2 were used for the first- and second-round amplifications, respectively. The cycling conditions were the same as those used for *Cryptosporidium* spp. (Table 1) (11). Positive and negative controls were included in each PCR assay. Positive amplicons were purified and subjected to Sanger sequencing. The obtained sequences were compared with reference sequences available in the NCBI database using BLAST,^2^ and species/genotype identification was further confirmed by phylogenetic analysis based on the obtained sequences and corresponding reference sequences.

**Table 1:** Primers and amplification conditions used for nested PCR detection and subtyping of *Cryptosporidium* spp. and *E. bieneusi*.

Parasite species	Gene locus	Primer sequences (5’-3’)	Fragment size (bp)	Annealing Temperature (°C)
*Cryptosporidium* spp.	SSU rRNA	CF2: GAC ATA TCA TTC AAG TTT CTG ACC CR2: CTG AAG GAG TAA GGA ACA ACC CF1: CCT ATC AGC TTT AGA CGG TAG G CR1: TCT AAG AAT TTC ACC TCT GAC TG	580	55
				55
	*gp60*	GP60F1: ATA GTC TCC GCT GTA TTC GP60R1: GGA AGG AAC GAT GTA TCT GP60F2: TCC GCT GTA TTC TCA GCC GP60R2: GCA GAG GAA CCA GCA TC	830	55
				55
*E. bieneusi*	ITS	ITSF1: GAT GGT CAT AGG GAT GAA GAG CTT ITSR1: TAT GCT TAA GTC CAG GGA G ITSF2: AGG GAT GAA GAG CTT CGG CTC TG ITSR2: AGT GAT CCT GTA TTA GGG ATA TT	400	55
				55

### 2.3 Haplotype network analysis and phylogenetic reconstruction

To characterize the genetic structure of *Cryptosporidium* spp. and *E. bieneusi* in horses from Inner Mongolia, relevant horse-derived sequences previously reported in China and other countries and regions were retrieved from the GenBank database. Multiple sequence alignments were performed using MEGA X (12).^3^ Haplotypes were subsequently identified using DnaSP. A haplotype network was constructed based on the statistical parsimony method with a 95% connection limit using TCS Networks (13). The network was further analyzed and visualized in PopART to illustrate haplotype composition and sample frequencies (14). Haplotypes were subsequently identified using DnaSP (15). Phylogenetic trees were constructed in MEGA X using the neighbor-joining method, with evolutionary distances estimated using the Kimura 2-parameter model. The reliability of tree topology was assessed using 1,000 bootstrap replicates.

### 2.4 Statistical analysis

Statistical analysis was performed using the chi-square test to calculate odds ratios (OR), 95% confidence intervals (CI), and *p* values to determine differences in prevalence among groups. Statistical significance was set at *p* < 0.05 for all comparisons. Analyses were performed using GraphPad Software Inc. version 8.02.

## 3 Results

### 3.1 Prevalence of *Cryptosporidium* spp. and *Enterocytozoon bieneusi*

A total of 325 horse fecal samples collected from Otog Banner, Zhenglan Banner, and Abaga Banner in Inner Mongolia were screened for *Cryptosporidium* spp. by nested PCR targeting the SSU rRNA gene. Nine samples were positive, yielding an overall prevalence of 2.8% (9/325) (Table 2). The prevalence of *Cryptosporidium* infection varied among the three regions, with the highest prevalence observed in Otog Banner at 6.7% (4/60), followed by Zhenglan Banner at 4.1% (5/121). No *Cryptosporidium*-positive samples were detected in Abaga Banner (0/144) (Table 2).

**Table 2:** Prevalence and molecular characterization of *Cryptosporidium* spp. and *E. bieneusi* in horses from the three sampling regions.

City	No. specimens	No. positive for *Cryptosporidium* (%)	*Cryptosporidium* species (no.)	*Cryptosporidiu*m subtype (no.)	No. positive for *E. bieneusi* (%)	*E. bieneusi* genotype (no.)
Otog Banner, Ordos City	60	4 (6.7)	*C. parvum* (4)		–	–
Zhenglan Banner, Xilingol League	121	5 (4.1)	*C. hominis* (5)	IkA18G1 (4), IkA19G1 (1)	5 (4.1)	horse 1 (5)
Abaga Banner, Xilingol League	144	–	–		–	–
Total	325	9 (2.8)	*C. parvum* (4) *C. hominis* (5)	IkA18G1 (4), IkA19G1 (1)	5 (1.5)	

ITS based detection of *E. bieneusi* identified five positive samples among the 325 horse fecal samples, corresponding to an overall prevalence of 1.5% (5/325) (Table 2). All five *E. bieneusi*-positive samples were detected in Zhenglan Banner, where the prevalence was 4.1% (5/121), whereas no positive samples were detected in Otog Banner or Abaga Banner (Table 2).

Further analysis according to age and health status revealed significant differences in prevalence. The prevalence of *Cryptosporidium* infection in foals was 11.5% (6/52), significantly higher than that in adult horses (1.1%, 3/273) (OR = 11.74, 95% CI: 3.059–43.50, *p* = 0.0007) (Table 3). In addition, the prevalence of *Cryptosporidium* infection was 16.1% (5/31) in diarrheic horses, which was significantly higher than that in non-diarrheic horses (1.4%, 4/294) (OR = 13.94, 95% CI: 3.779–46.84, *p* = 0.0006; Table 3). In contrast, no significant differences in the prevalence of *E. bieneusi* were observed among groups stratified by age or health status.

**Table 3:** Association of age and health status with *Cryptosporidium* spp. and *E. bieneusi* infection in horses.

Pathogen	Variable	Groups	No. tested	No. positive	OR (95% CI)	*p*-value
*Cryptosporidium* spp.	Age	Foal	52	6	11.74 (3.059–43.50)	*p* = 0.0007
		Adult	273	3		
	Health status	Diarrheic	31	5	13.94 (3.779–46.84)	*p* = 0.0006
		Non-diarrheic	294	4		
*E. bieneusi*	Age	Foal	52	1	1.32 (0.14–12.04)	*p* = 0.584
		Adult	273	4		
	Health status	Diarrheic	31	2	6.69 (1.07–41.68)	*p* = 0.073
		Non-diarrheic	294	3		

### 3.2 *Cryptosporidium* species and subtypes and *Enterocytozoon bieneusi* genotypes

Based on sequence analysis of an approximately 580-bp fragment of the SSU rRNA gene from the nine *Cryptosporidium*-positive samples, two species were identified: *C. hominis* (55.6%, 5/9) and *C. parvum* (44.4%, 4/9). *gp60* amplicons were successfully obtained from all five *C. hominis*-positive samples, whereas amplification was unsuccessful for the four *C. parvum*-positive samples. Sequence analysis of the *C. hominis* amplicons identified four isolates as IkA18G1 and one as IkA19G1. Sequence analysis of an approximately 242-bp ITS sequence from the five *E. bieneusi*-positive samples identified all isolates as genotype horse1, accounting for 100% (5/5) of the positive samples.

The identified species and genotypes showed different distributions among the three sampling regions. All five *C. hominis*-positive samples were detected in Zhenglan Banner, whereas all four *C. parvum*-positive samples were detected in Otog Banner. The *E. bieneusi* horse1 genotype was detected only in Zhenglan Banner. No *Cryptosporidium*- or *E. bieneusi*-positive samples were detected in Abaga Banner.

### 3.3 Haplotype network and phylogenetic analysis

The SSU rRNA gene sequences of *C. hominis* obtained in this study were combined with previously published horse- and donkey-derived sequences from other regions available in GenBank to construct a phylogenetic tree and haplotype network.

Phylogenetic analysis showed that the sequences obtained in this study clustered into two distinct branches. Sequences PZ299408, PZ299409, and PZ299412 clustered in the same branch as the donkey-derived sequence MK775038 from Hohhot, China, with sequence similarities ranging from 97.7 to 99.4% (Figure 2A; Table 4). Sequences PZ299410 and PZ299411 clustered in another branch with the horse-derived sequence PP663681 from Anhui, China, showing sequence similarities of 98.1 and 96.9%, respectively (Figure 2A; Table 4). In addition, the *C. hominis* subtypes IkA18G1 and IkA19G1 identified in this study clustered together with other Ik subtypes, forming a distinct Ik subtype clade (Figure 2B). Haplotype analysis identified a total of eight haplotypes. Hap_6, Hap_7, and Hap_8 were identified in sequences from other regions of China, whereas Hap_6 and Hap_7 were identified in sequences from other countries. Five haplotypes were identified among the sequences obtained in the present study, namely Hap_1, Hap_2, Hap_3, Hap_4, and Hap_5. Hap_7 was the most common haplotype, accounting for 33.3% of all sequences, but was not detected among the sequences obtained in this study. Hap_1–Hap_5 differed from Hap_7 by 5–11 bp (Figure 2C).

**Figure 2:**
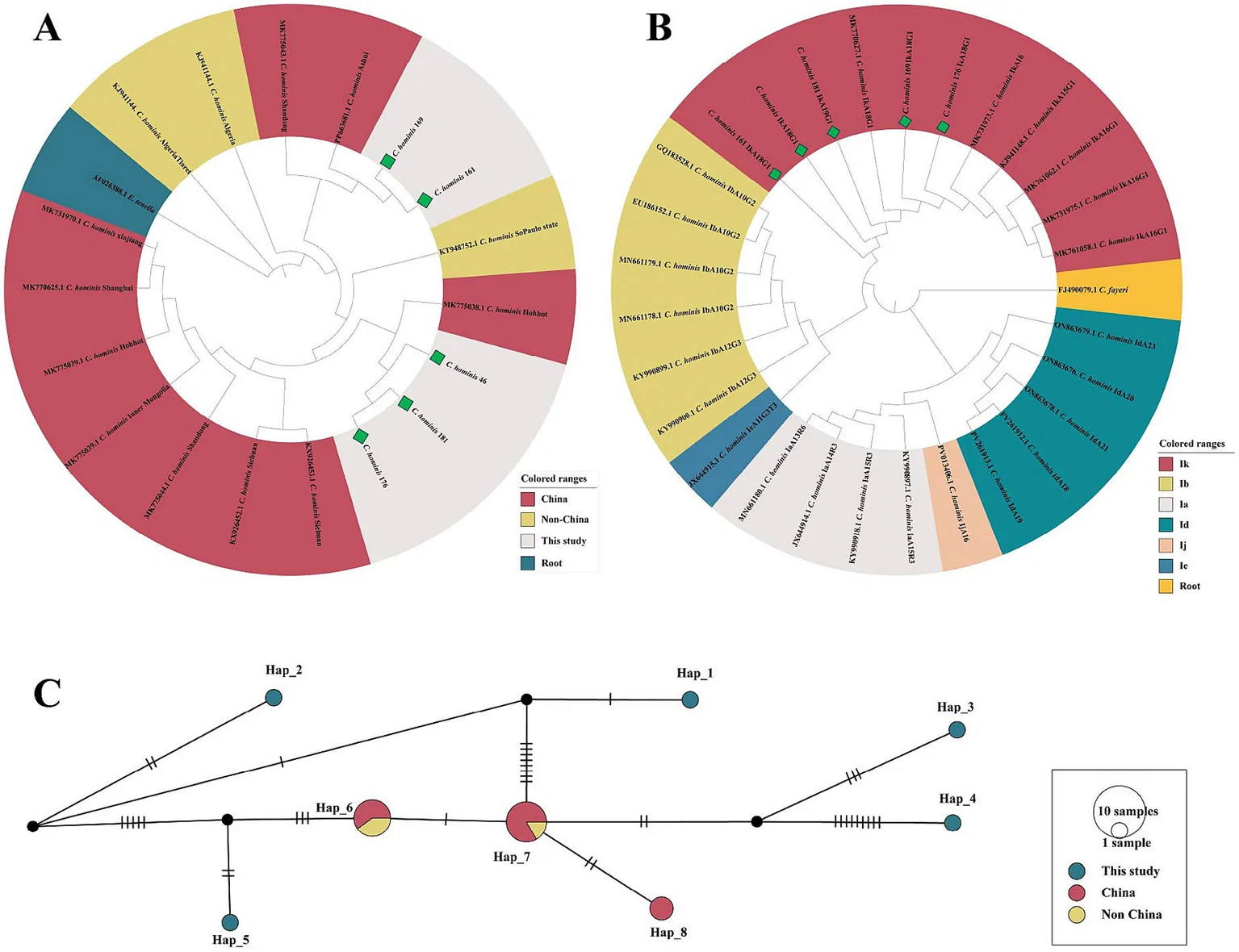
Phylogenetic tree and haplotype analysis of *C. hominis* **(A)** Phylogenetic tree of the *C. hominis* SSU rRNA gene. The tree was constructed using the neighbor-joining method with the Kimura 2-parameter model in MEGA X. **(B)** Phylogenetic tree of *C. hominis* gp6*0* gene. The tree was constructed using the neighbor-joining method with the Kimura 2-parameter model in MEGA X. **(C)** Haplotype analysis results of *C. hominis* sequences detected in this study and published *C. hominis* sequences. Different colors represent different groups, including this study, China and Non-China, and the sizes of the circles represent the number of haplotypes.

**Table 4:** Sequence similarity analysis of *Cryptosporidium* SSU rRNA and *E. bieneusi* ITS sequences obtained in this study.

Species	Accession number (Sample number)	Similarity (%)	Reference accession no. /host/location
*C. hominis*	PZ299408.1 (176)	98	MK775038.1- donkey - China Hohhot
	PZ299409.1 (181)	97.7	MK775038.1- donkey - China Hohhot
	PZ299410.1 (161)	98.1	PP663681.1- horse - China Anhui
	PZ299411.1 (169)	96.9	PP663681.1- horse - China Anhui
	PZ299412.1 (46)	99.4	MK775038.1- donkey - China Hohhot
*C. parvum*	PZ299405.1 (26)	99.8	KU200953.1- donkey - China Inner Mongolia
	PZ748770.1 (42)	99.5	KU200953.1- donkey - China Inner Mongolia
	PZ299406.1 (4)	99.1	KU200953.1- donkey - China Inner Mongolia
	PZ299407.1 (39)	100	KU200953.1- donkey - China Inner Mongolia
*E. bieneusi*	PZ575365.1 (164)	99.72	MN704924.1- donkey - China
	PZ575366.1 (175)	99.45	MN174115.1- donkey - China
	PZ575367.1 (169)	99.45	KX905206.1 - rhesus macaque - China
	PZ575368.1 (115)	99.72	KX905206.1 - rhesus macaque - China

Phylogenetic analysis of *C. parvum* showed that the four SSU rRNA sequences identified in this study clustered in the same branch as the donkey-derived sequence KU200953 from Inner Mongolia, China, with sequence similarities ranging from 99.1 to 100% (Figure 3A; Table 4). Haplotype analysis identified a total of five haplotypes. Hap_1 and Hap_4 were identified in sequences from other regions of China, whereas Hap_1 and Hap_5 were detected in sequences from other countries. Three haplotypes were identified among the sequences obtained in this study, namely Hap_1, Hap_2, and Hap_3. Hap_1 was the most common haplotype, accounting for 76.5% of all sequences. Hap_2 and Hap_3 differed from Hap_1 by three and one nucleotide substitutions, respectively (Figure 3B).

**Figure 3:**
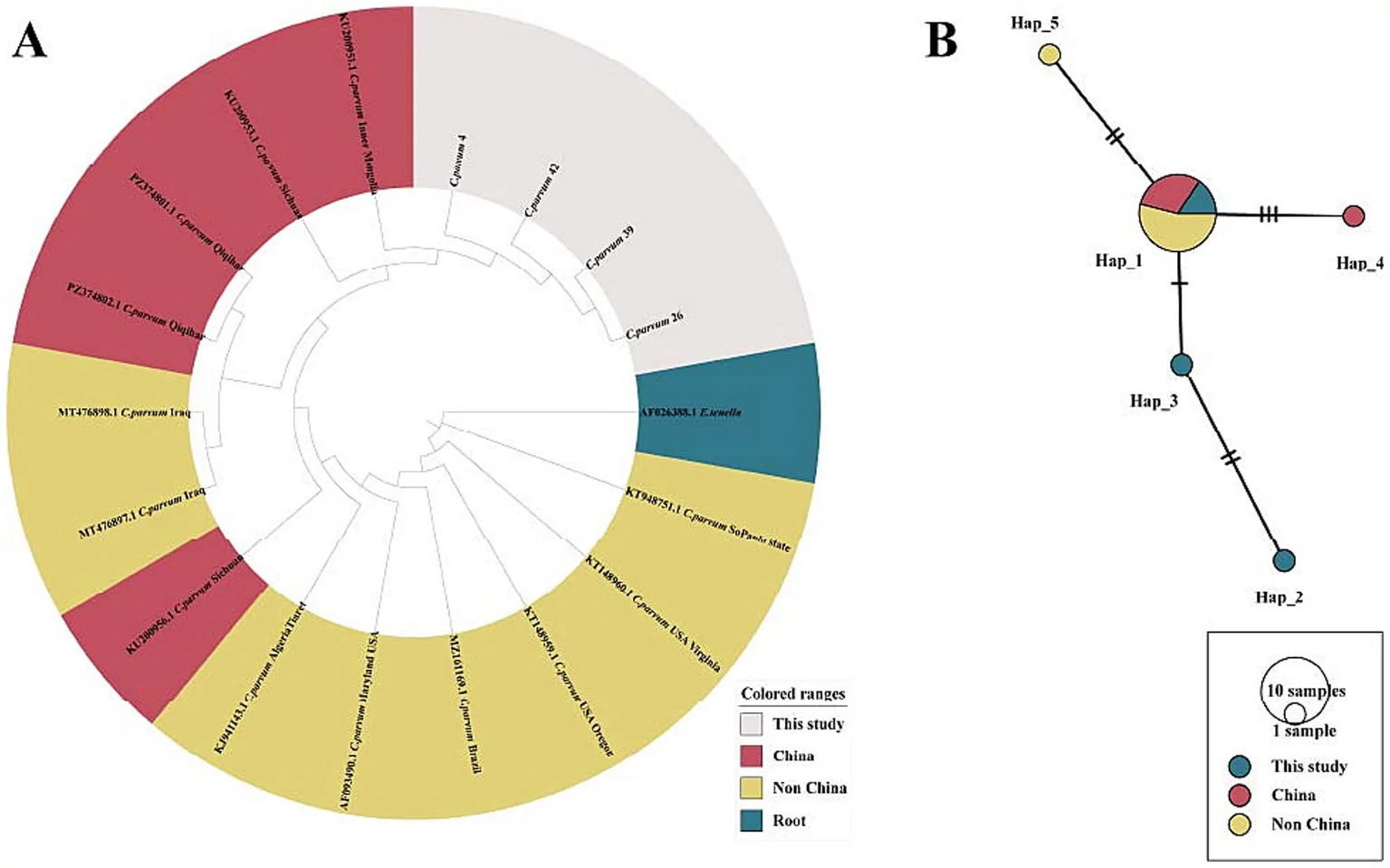
Phylogenetic tree and haplotype analysis of *C. parvum*. **(A)** Phylogenetic tree of the *C. parvum* SSU rRNA gene. The tree was constructed using the neighbor-joining method with the Kimura 2-parameter model in MEGA X. **(B)** Haplotype analysis results of *C. parvum* sequences detected in this study and published *C. parvum* sequences. Different colors represent different groups, including this study, China, and Non-China, and the sizes of the circles represent the number of haplotypes.

Phylogenetic and haplotype network analyses of the 242 bp ITS sequences of *E. bieneusi* revealed the genetic relationships and distribution patterns of *E. bieneusi* in horses from Inner Mongolia. All isolates clustered within Group 1, a clade that has been considered to have high zoonotic potential in previous studies (Figure 4A; Table 4). Haplotype analysis of the horse1 genotype identified two haplotypes. Hap_1 and Hap_2 were detected in sequences from China, whereas only Hap_1 was identified in sequences from other countries. All sequences obtained in the present study belonged to Hap_1. Hap_1 was the most common haplotype, accounting for 93.8% of all sequences, and differed from Hap_2 at eight nucleotide positions (Figure 4B).

**Figure 4:**
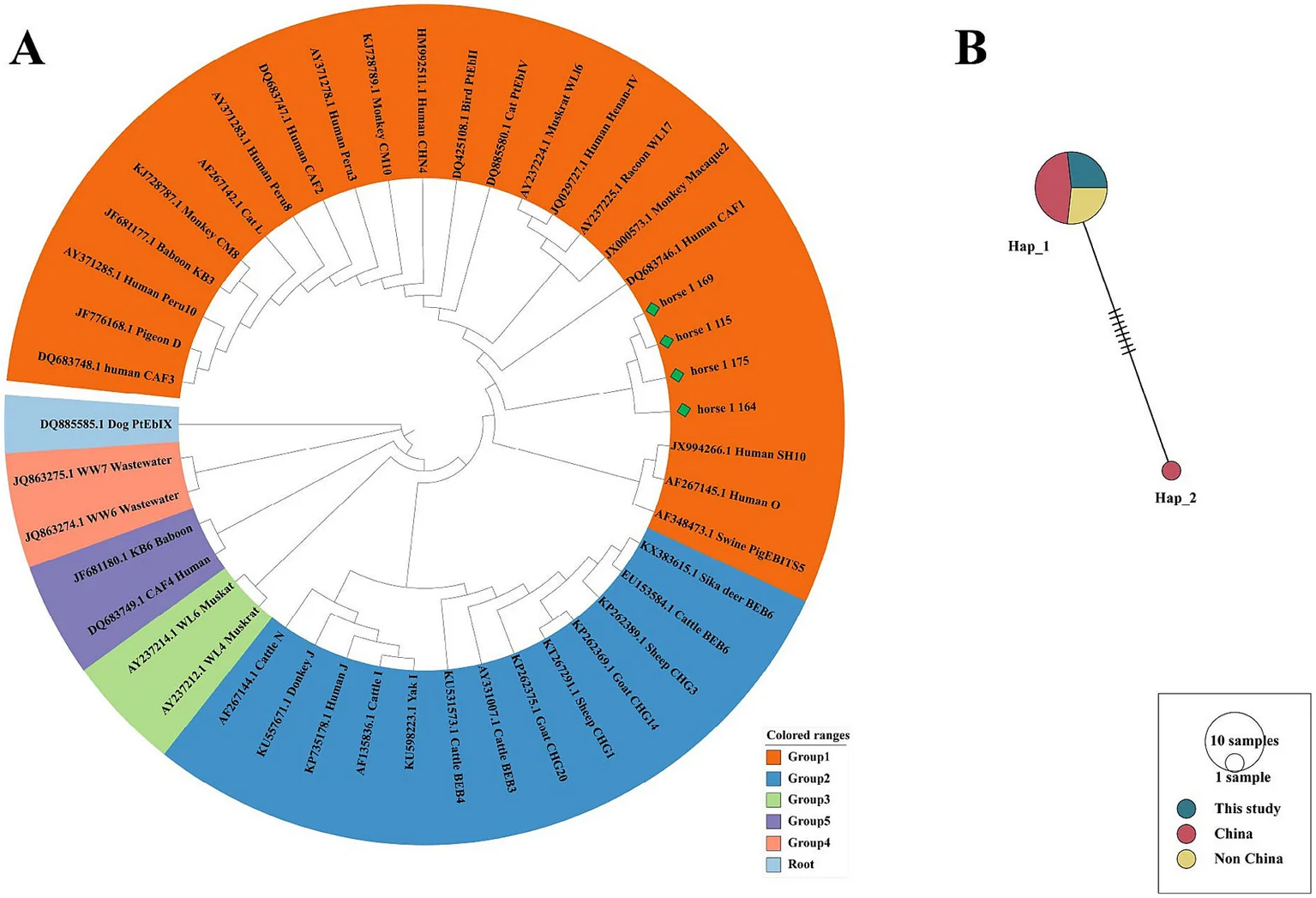
Phylogenetic tree and haplotype analysis of *E. bieneusi*. **(A)** Phylogenetic tree of the *E. bieneusi* ITS region. The tree was constructed using the Neighbor-joining method with the Kimura 2-parameter model in MEGA X. **(B)** Haplotype analysis results of *E. bieneusi* sequences detected in this study and published *E. bieneusi* sequences. Different colors represent different groups, including this study, China and Non-China, and the sizes of the circles represent the number of haplotypes.

## 4 Discussion

The present study systematically investigated the prevalence and genetic characteristics of *Cryptosporidium* spp. and *E. bieneusi* in horses in Inner Mongolia. The findings suggest that some of the species and genotypes identified in this study have potential zoonotic significance. These findings provide additional molecular epidemiological and genetic data on *Cryptosporidium* spp. and *E. bieneusi* in horses in Inner Mongolia and further improve our understanding of the prevalence and genetic characteristics of these two pathogens in horse populations. These findings also provide baseline information for assessing the potential public health risks associated with horse-derived *Cryptosporidium* spp. and *E. bieneusi* and for developing targeted surveillance strategies.

The results of the present study showed that *Cryptosporidium* spp. and *E. bieneusi* infections were present in horses in Inner Mongolia, with prevalence rates differing to some extent from those reported in previous studies. The overall prevalence of *Cryptosporidium* spp. was 2.8%, which was comparable to the 3.1% (10/327) reported in horses from Shandong, Xinjiang, and Inner Mongolia (8), higher than the 1.8% (11/621) reported in Chinese racehorses (16), but lower than the recent estimate of 4.1% (17/412) in horses from northern and eastern China (17). The overall prevalence of *E. bieneusi* was 1.5%, which was lower than the 22.5% (75/333) reported in horses from southwestern China (18), 30.9% (81/262) in grazing horses from Xinjiang (19), 4.8% (30/621) in Chinese racehorses (20), and 7.4% (24/325) in horses from northern China (9). Differences in prevalence among studies may be associated with factors such as geographic and environmental conditions, the age structure of horse populations, husbandry and management practices, sampling periods, and diagnostic methods (21). Further analysis of host-related factors showed that the prevalence of *Cryptosporidium* infection was significantly higher in foals than in adult horses (11.5% vs. 1.1%), consistent with previous reports indicating a higher risk of infection in young horses (17). This age-related difference may reflect the greater susceptibility of foals to *Cryptosporidium* infection. As horses age, maturation of host immune defenses, together with partial protection acquired through previous natural exposure, may reduce the likelihood of subsequent infection (22). Furthermore, the regional differences in the distribution of these two pathogens observed in the present study may be associated with variations in the age composition of horses across the sampling regions. Given that most positive samples were obtained from foals and that a substantial proportion of the sampled foals originated from Zhenglan Banner, the higher detection frequency in this region may, at least in part, reflect differences in the age structure of the sampled populations. Meanwhile, the prevalence of *Cryptosporidium* infection was significantly higher in diarrheic horses than in non-diarrheic horses (16.1% vs. 1.4%), suggesting a possible association between *Cryptosporidium* infection and diarrhea in horses. *Cryptosporidium* infection can damage the intestinal epithelium and impair normal digestive and absorptive functions, which may contribute to the development of diarrhea and other gastrointestinal symptoms in infected animals (23). In contrast, no significant associations were observed between *E. bieneusi* prevalence and either age or health status in the present study. Taken together, age and health status may be important factors associated with *Cryptosporidium* infection in horses, and particular attention should be given to young horses and diarrheic individuals during surveillance and control programs.

Further species and subtype characterization of *Cryptosporidium* spp. revealed the presence of species and subtypes with potential zoonotic risk in horses in Inner Mongolia. Two *Cryptosporidium* species, *C. hominis* and *C. parvum*, were identified in the present study. *C. hominis* is primarily associated with human infections but has also been reported in animal hosts such as horses and donkeys (24), whereas *C. parvum* is an important zoonotic species that can infect humans and a wide range of livestock species (25). Both species have previously been reported in horses in China. Further *gp60* subtyping showed that all *C. hominis* isolates identified in this study belonged to the Ik subtype family, including IkA18G1 and IkA19G1. Notably, this study represents the first report of the *C. hominis gp60* subtype IkA19G1 in Inner Mongolia. Previous studies have identified IkA18G1 in Chinese racehorses and IkA19G1 in Bactrian camels from Xinjiang. In addition, Ik subtypes have also been reported in human infections, indicating that this subtype family occurs in multiple host species and may have zoonotic potential (16, 26, 27). In addition to subtype composition, haplotype analysis of the SSU rRNA sequences showed that the *C. hominis* isolates identified in this study belonged to different haplotypes and exhibited several nucleotide differences from the predominant haplotypes previously reported in China and other countries, suggesting a certain degree of genetic diversity among *C. hominis* isolates from horses in Inner Mongolia. Notably, although all of these isolates belonged to the Ik subtype family at the *gp60* locus, sequence variation was still observed at the SSU rRNA locus, suggesting a certain degree of genetic heterogeneity within the same subtype family. Overall, this study revealed the species composition, subtype characteristics, and genetic diversity of *Cryptosporidium* spp. in horses in Inner Mongolia, further indicating the potential zoonotic risk of horse-derived *Cryptosporidium*. These findings provide important reference information for strengthening molecular surveillance in horse populations and evaluating the public health significance of these parasites.

Genotyping and genetic analyses of *E. bieneusi* further revealed the genetic composition of horse-derived isolates in the study area. All five *E. bieneusi*-positive samples identified in this study belonged to the horse1 genotype, suggesting that horse1 may represent a predominant genotype circulating in horses in the study area. The horse1 genotype is relatively common in equids and has previously been detected in horses from Xinjiang, southwestern China, and Chinese racehorse populations. It has also been reported in horses from Colombia, Algeria, and the Czech Republic, indicating a broad geographic distribution (7, 18–20, 28, 29). Phylogenetic analysis showed that all horse1 sequences obtained in this study clustered within Group 1, which contains numerous genotypes with broad host ranges and potential zoonotic significance, suggesting that the horse1 genotype may also be of public health importance. Haplotype network analysis further showed that all horse1 sequences obtained in this study belonged to Hap_1. This haplotype was also present in reference sequences from China and other countries and accounted for 93.8% of all sequences analyzed, indicating that horse1 has a relatively conserved genetic structure and that Hap_1 may represent its predominant and widely distributed haplotype. Taken together, these findings suggest that the horse1 genotype may exhibit relatively stable circulation and persistence in horse populations across different regions. Its placement within Group 1, which has relatively high zoonotic potential, further highlights the need to investigate the possible transmission risk of horse-derived *E. bieneusi* at the human–animal interface.

This study has several limitations, including the limited geographic coverage and sample size, the lack of long-term longitudinal surveillance, and the failure to obtain further gp60 subtype information for *C. parvum*. One limitation of this study is that potential clustering effects associated with sampling from different farms and regions were not considered, which may have affected the interpretation of the observed associations. Future studies should include larger and more balanced sample sizes across farms and regions and apply multivariable or mixed-effects models to account for potential clustering and confounding effects.

## 5 Conclusion

This study revealed the prevalence and genetic characteristics of *Cryptosporidium* spp. and *E. bieneusi* in horses in Inner Mongolia. Molecular analyses identified two *Cryptosporidium* species (*C. hominis* and *C. parvum*) and the *E. bieneusi* genotype horse1. Genetic analyses revealed variation among *C. hominis* isolates, including the identification of the *gp60* subtype IkA19G1, which is reported for the first time in Inner Mongolia. The significantly higher prevalence of *Cryptosporidium* infection in foals and diarrheic horses highlights the importance of targeted surveillance in these groups. Overall, this study adds to the available molecular epidemiological data on these two enteric pathogens in horses in Inner Mongolia and indicates their potential zoonotic significance, providing baseline information for future molecular surveillance and assessment of possible zoonotic transmission risks.

## Data Availability

The datasets presented in this study can be found in online repositories. The names of the repository/repositories and accession number(s) can be found at: https://www.ncbi.nlm.nih.gov/genbank/, PZ299405–PZ299412, PZ748770, PZ575365–PZ575368.

## Footnotes

1
https://www.moa.gov.cn/

2
https://blast.ncbi.nlm.nih.gov/Blast.cgi

3
www.megasoftware.net
